# BiG-MAP: an Automated Pipeline To Profile Metabolic Gene Cluster Abundance and Expression in Microbiomes

**DOI:** 10.1128/mSystems.00937-21

**Published:** 2021-09-28

**Authors:** Victória Pascal Andreu, Hannah E. Augustijn, Koen van den Berg, Justin J. J. van der Hooft, Michael A. Fischbach, Marnix H. Medema

**Affiliations:** a Bioinformatics Group, Wageningen University, Wageningen, the Netherlands; b Department of Bioengineering, Stanford University, Stanford, California, USA; c Department of Microbiology and Immunology, Stanford University, Stanford, California, USA; d ChEM-H, Stanford University, Stanford, California, USA; University of Massachusetts Medical School

**Keywords:** metabolic gene cluster, metagenomics, metatranscriptomics, microbiome-associated phenotype, biosynthesis, metabolomics, microbiome, specialized metabolism

## Abstract

Microbial gene clusters encoding the biosynthesis of primary and secondary metabolites play key roles in shaping microbial ecosystems and driving microbiome-associated phenotypes. Although effective approaches exist to evaluate the metabolic potential of such bacteria through identification of these metabolic gene clusters in their genomes, no automated pipelines exist to profile the abundance and expression levels of such gene clusters in microbiome samples to generate hypotheses about their functional roles, and to find associations with phenotypes of interest. Here, we describe BiG-MAP, a bioinformatic tool to profile abundance and expression levels of gene clusters across metagenomic and metatranscriptomic data and evaluate their differential abundance and expression under different conditions. To illustrate its usefulness, we analyzed 96 metagenomic samples from healthy and caries-associated human oral microbiome samples and identified 252 gene clusters, including unreported ones, that were significantly more abundant in either phenotype. Among them, we found the *muc* operon, a gene cluster known to be associated with tooth decay. Additionally, we found a putative reuterin biosynthetic gene cluster from a Streptococcus strain to be enriched but not exclusively found in healthy samples; metabolomic data from the same samples showed masses with fragmentation patterns consistent with (poly)acrolein, which is known to spontaneously form from the products of the reuterin pathway and has been previously shown to inhibit pathogenic Streptococcus mutans strains. Thus, we show how BiG-MAP can be used to generate new hypotheses on potential drivers of microbiome-associated phenotypes and prioritize the experimental characterization of relevant gene clusters that may mediate them.

**IMPORTANCE** Microbes play an increasingly recognized role in determining host-associated phenotypes by producing small molecules that interact with other microorganisms or host cells. The production of these molecules is often encoded in syntenic genomic regions, also known as gene clusters. With the increasing numbers of (multi)omics data sets that can help in understanding complex ecosystems at a much deeper level, there is a need to create tools that can automate the process of analyzing these gene clusters across omics data sets. This report presents a new software tool called BiG-MAP, which allows assessing gene cluster abundance and expression in microbiome samples using metagenomic and metatranscriptomic data. Here, we describe the tool and its functionalities, as well as its validation using a mock community. Finally, using an oral microbiome data set, we show how it can be used to generate hypotheses regarding the functional roles of gene clusters in mediating host phenotypes.

## INTRODUCTION

Bacteria can produce diverse sets of small molecules that interact with other microbes or with their host. These metabolites include members of both primary and secondary metabolism and cover a wide chemical diversity (1, 2). Importantly, the pathways responsible for their production are often specific to certain strains or species and help them to compete for space and resources (3), e.g., through antimicrobial, nutrient-scavenging, or immunomodulatory activities (4). The genes that encode these pathways are often physically clustered and are also known as biosynthetic gene clusters (BGCs) or metabolic gene clusters (MGCs) (5, 6)—the latter having a broader definition that also includes catabolic and energy-generating pathways. Several studies have indicated metabolites produced from such gene clusters to be the major drivers of specific phenotypic traits; for instance, pseudomonads in the rhizosphere of sugar beet plants were shown to produce the antifungal nonribosomal peptide (NRP) thanamycin, which protects plants from fungal infections (7). Another example from primary metabolism is trimethylamine, a diet-derived molecule that is processed by bacteria harboring the *cut* gene cluster and that has been associated with an increased risk of cardiovascular disease (8). Therefore, mining genomes for MGCs enables a deeper understanding of function at the molecular level and can help to determine the role a given microbe plays in the ecosystem (9).

Several tools have been developed to mine genomes for these gene clusters, including antiSMASH (10), gutSMASH (11), and DeepBGC (12). In contrast to other tools for functional profiling of microbial communities, such as HUMAnN2 (13), MetaPath (14), FMAP (15), and Metatrans (16), these do not depend on pathways that are present in reference databases like KEGG (17) or MetaCyc (18), which include only pathways for which most or all enzymatic steps have been elucidated. In fact, the majority of gene clusters identified by antiSMASH and many gene clusters predicted by gutSMASH encode pathways for which the catalytic steps, intermediates, and final products are unknown. However, known pathways that are encoded by gene clusters can also be reliably detected. The detection of complete gene clusters instead of individual enzyme-coding genes likely decreases false-positive detections of enzymes that show sequence similarity to reference enzyme sequences but are part of different functional contexts. For these reasons, identification of gene clusters of known and unknown function provides a useful basis to look for functional explanations of microbiome-associated phenotypes of interest. As phenotypes are often triggered by metabolites at physiologically relevant concentrations, while samples without the phenotype lack these metabolites or have them at lower concentrations, assessing gene cluster abundance and expression levels across samples is crucial to predict associations with the phenotype in question. Another significant advantage of profiling the community by combining different omics data is to prioritize the characterization of putative gene clusters that are highly abundant or expressed in samples of interest, in order to elucidate the structures and functions of the most relevant novel compounds and their biosynthetic pathways.

Here, we present BiG-MAP (the Biosynthetic Gene cluster Meta’omics Abundance Profiler), which provides a streamlined and automated process to determine BGC/MGC abundance and expression in bacterial communities by mapping metagenomic and metatranscriptomic reads to gene cluster sequences from reference genomes or metagenomic assemblies. BiG-MAP uses MinHash-based redundancy filtering to avoid ambiguous mapping, groups BGCs into gene cluster families (GCFs) with BiG-SCAPE (19), and uses these to output and visualize profiles of MGC or GCF abundance or expression levels across samples. Additionally, it calculates differential abundance or expression using either parametric or nonparametric tests. We validated the tool using simulated metagenomic data and show how MGC abundance and expression levels are accurately recapitulated. Finally, to showcase its usefulness, we applied BiG-MAP on a large publicly available metagenome data set from the human oral microbiome and describe how it successfully identified differential abundance of gene clusters related to bacterial specialized primary and secondary metabolism that are (potentially) relevant for caries development. Among others, this collection includes a *pdu* and cobalamin gene cluster involved in reuterin biosynthesis and the *muc* operon involved in reutericyclin/mutanocyclin biosynthesis. Thus, BiG-MAP yields novel insights into the onset and development of oral cavities.

## RESULTS AND DISCUSSION

### An approach to map metagenomics and metatranscriptomic reads to gene clusters.

BiG-MAP maps shotgun sequencing reads onto gene clusters that have been predicted by either antiSMASH (10) or gutSMASH (11). It is a Python-based pipeline, which allows downloading data sets from the Sequence Read Archive (SRA), mapping metagenomic or metatranscriptomic reads to gene clusters detected in reference genome collections or in a metagenomic assembly, providing normalized counts across samples, performing differential analyses, and visualizing the results. It requires three main inputs: (i) a gene cluster collection obtained from running any “SMASH-based” algorithm, (ii) the meta-omic data set in FASTQ or FASTA format or, alternatively, the SRA accession numbers to download it, and (iii) a metadata file with sample information to segregate them into groups and compare their gene cluster contents. The minimal requirements for the metagenomic and metatranscriptomic data sets for a meaningful analysis using BiG-MAP depend on various factors. Naturally, at least three samples (and ideally considerably more) for each phenotype are required when performing a differential abundance analysis between phenotypes. The required sequencing depth per sample will depend on the research question: does one want to study mainly expression patterns for MGCs from highly abundant community members or also for those of rare members? The user should pay attention in the experimental design that sufficient observations (i.e., mapped reads, which will depend on the coverage per MGC) are present across samples to test for significant differences between conditions. When using BiG-MAP, the user has the choice to map metagenomic or metatranscriptomic reads either to MGC sequences from a set of representative reference genomes or to MGCs detected in a metagenomic assembly originating from the same data or the same biological samples. In the latter case, additional steps (quality control and assembly) are required to assemble the data before using gutSMASH and/or antiSMASH to detect MGCs and before using BiG-MAP to map reads against them.

**FIG 1:**
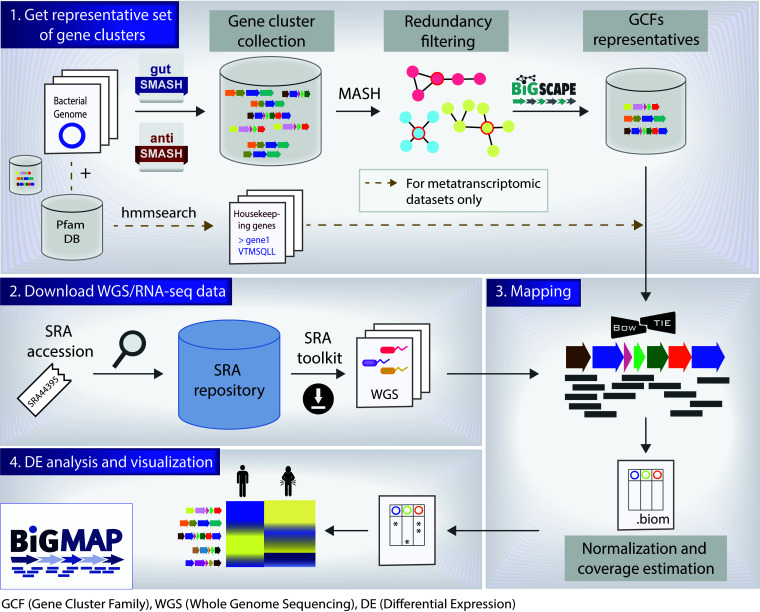
BiG-MAP workflow. BiG-MAP is composed of four different modules: (1) BiG-MAP.family, which, given a set of predicted gene clusters by either gutSMASH or antiSMASH, returns a representative set of nonredundant gene clusters based on sequence similarity (this module also identifies the protein sequences of 5 housekeeping genes from the bacterial genomes that encode the representative gene clusters when metatranscriptomes are used); (2) BiG-MAP.download, to download a set of metagenomes/metatranscriptomes given their SRA accessions; (3) BiG-MAP.map, to align omics reads to the representative set of gene clusters using Bowtie; and (4) BiG-MAP.analyse, to normalize and perform differential abundance/expression analysis of gene clusters across different conditions and visualize the results (see Fig. S1 and S2 as an example).

BiG-MAP is composed of four different modules (Fig. 1): (i) BiG-MAP.family, which performs redundancy filtering on the input collection of predicted gene clusters and provides a set of representative gene clusters for the mapping process; (ii) BiG-MAP.download, which uses a list of SRA accession numbers to download the shotgun data if present in the SRA database (this step is optional); (iii) BiG-MAP.map, which maps reads from the metagenomic or metatranscriptomic samples onto the set of representative gene clusters obtained from BiG-MAP.family; and (iv) BiG-MAP.analyse, which normalizes the counts for sparsity and sequencing depth, performs differential abundance/expression analysis, and visualizes the output.

10.1128/mSystems.00937-21.2 FIG S1 Illustration of BiG-MAP.analyse module output when using metagenomics data. In this case, for testing and developing purposes, the CGR genomes were used as input for gutSMASH. The resulting predictions were used as input together with the metagenomics samples from Schirmer et al. (M. Schirmer, E. A. Franzosa, J. Lloyd-Price, L. J. McIver, R. Schwager, T. W. Poon, A. N. Ananthakrishnan, E. Andrews, G. Barron, K. Lake, M. Prasad, J. Sauk, B. Stevens, R. G. Wilson, J. Braun, L. A. Denson, S. Kugathasan, D. P. B. McGovern, H. Vlamakis, R. J. Xavier, and C. Huttenhower, Nat Microbiol 3:337–346, 2018, https://doi.org/10.1038/s41564-017-0089-z) (PRJNA389280). The significant differential abundances of 11 gene clusters across Crohn’s disease (CD) samples and healthy (non-IBD) samples using Kruskal Wallis are shown in the heatmap. Phenotypes (CD or non-IBD) are shown in a bar on top of the heatmap. The more abundant a gene cluster is, the more yellow it is shown in the heatmap. Next to the heatmap, the bar chart represents the abundance log_2_ fold change values, which indicate the overall difference in abundance of an MGC between the phenotypes across all samples. On the far right, the dots represent the coverage values computed by BiG-MAP to show what proportion of the whole gene cluster has been covered by reads, in blue for the CD samples and in orange for the healthy samples. Download FIG S1, TIF file, 1.1 MB. Copyright © 2021 Pascal Andreu et al. 2021 Pascal Andreu et al. https://creativecommons.org/licenses/by/4.0/ This content is distributed under the terms of the Creative Commons Attribution 4.0 International license.

10.1128/mSystems.00937-21.3 FIG S2 Illustration of BiG-MAP analysis module output when using metatranscriptomic data. In this case, for testing and developing purposes, the CGR genomes were used as input for gutSMASH. The resulting predictions were used as input together with the metatranscriptomes from Schirmer et al. The significant differential expression of 8 gene clusters across CD samples and healthy (non-IBD) using the Kruskal-Wallis test are displayed in the heatmap. The higher the expression of a gene cluster is, the more yellow it is colored in the heatmap. Similar to Fig. S1, the log_2_ fold change and coverage values are also depicted. When analyzing metatranscriptomic data, a heatmap with the expression values of five housekeeping genes is also included, which is used to set an expression baseline. Download FIG S2, TIF file, 1.2 MB. Copyright © 2021 Pascal Andreu et al. 2021 Pascal Andreu et al. https://creativecommons.org/licenses/by/4.0/ This content is distributed under the terms of the Creative Commons Attribution 4.0 International license.

The BiG-MAP.family module performs a redundancy analysis on the gene cluster collection to remove almost-identical sequences, in order to reduce the computing time and avoid problems with ambiguous read mapping. To achieve this, the protein sequences encoded in each of the gene clusters are used as input for MASH (20), a MinHash-based algorithm to estimate sequence distance. Next, a representative gene cluster is selected based on a medoid calculation. The resulting representatives are then clustered into GCFs using BiG-SCAPE (19), an algorithm that uses three different distance metrics to group MGCs into families based on sequence and architectural similarity. This step helps to group more distantly related homologous gene clusters that likely have the same chemical products but that are encoded in more distantly related organisms. In such cases, BiG-MAP maps reads to the family representatives separately, but it also allows reporting combined abundance or expression levels per family to find associations with phenotypes at a higher level. In order to set an expression baseline when using metatranscriptomic data, BiG-MAP screens bacterial genomes whose gene clusters have been included in the nonredundant representative set of gene clusters for five housekeeping genes known to have stable expression levels using HMMer (for details, see Materials and Methods section titled “BiG-MAP.family: creating a nonredundant MGC representative collection”). Next, the reads are mapped to the representative gene clusters using the short-read aligner Bowtie2 (21). The obtained raw read counts are then converted to RPKM (reads per kilobase per million) values, which are summed across all representative MGCs within a GCF when reporting family abundances (raw counts are also output for each representative MGC). In the last module, BiG-MAP.analyse, RPKM values are then normalized using cumulative sum scaling (22) (CSS) to account for sparsity. Moreover, for each aligned gene cluster, BiG-MAP assesses read coverage to control for gene clusters that are only partially mapped to by meta-omic reads. BiG-MAP reports two coverage values in the intermediate files, one for the whole gene cluster and the other considering only the core genes of the BGC/MGC; both numbers are useful in cases where gene cluster boundaries called by antiSMASH or gutSMASH are imprecise and reads may be mapped to regions flanking the actual gene cluster. Subsequently, BiG-MAP detects differentially abundant or differentially expressed gene clusters by using either zero-inflated Gaussian distribution mixture models (ZIG models) or using a Kruskal-Wallis model. Finally, all the generated results are displayed in a plot that includes a heatmap for the gene cluster abundance/expression values, a bar plot for the log fold change, the coverage values, and, finally, another heatmap for the housekeeping gene expression values when analyzing metatranscriptomes (see Fig. S1 to S3 in the supplemental material). The output folders contain various intermediate and final results, including the BiG-SCAPE results, the resulting bedgraphs, the raw and normalized RPKM counts for each sample (in BIOM format [23]), the results of the fitZIG and Kruskal-Wallis tests in tab-separated tables, and mapping coverage values for each gene cluster and sample. Altogether, this tool presents a streamlined method to functionally profile meta-omics data by mapping reads to known or putative gene clusters.

### Assessing and validating BiG-MAP performance using simulated data.

**FIG 2:**
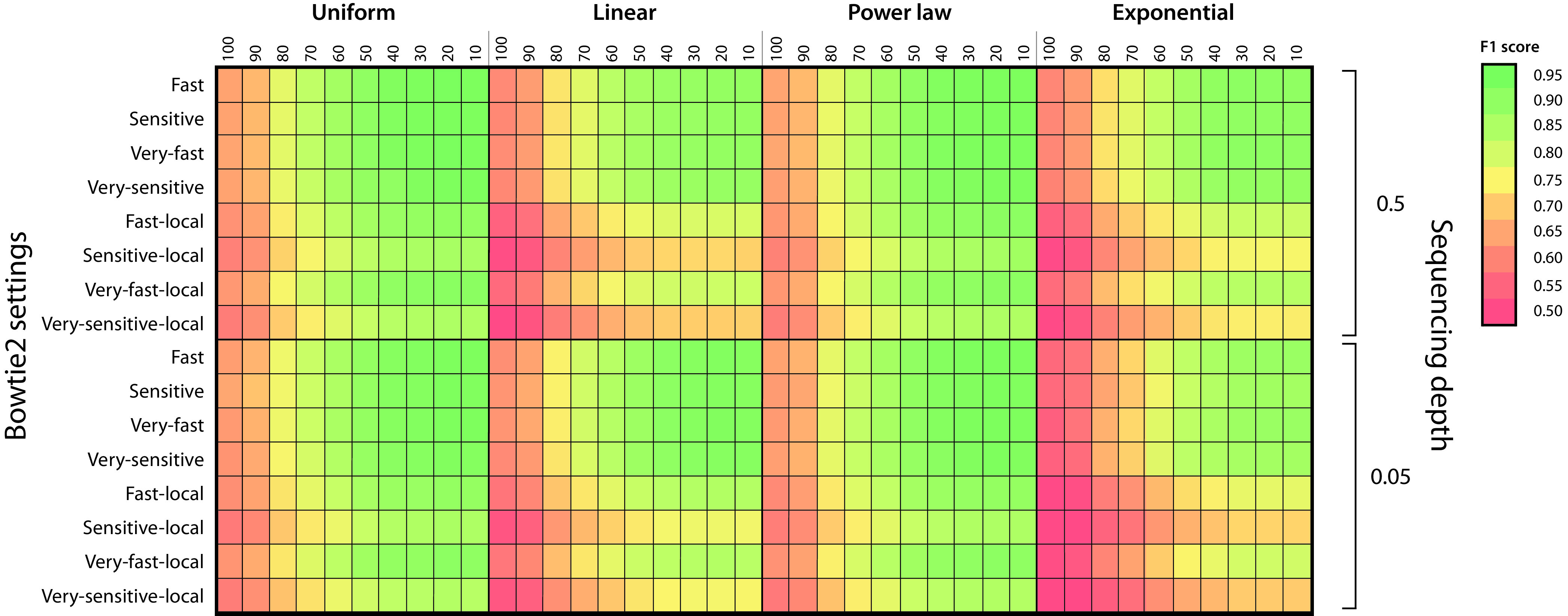
BiG-MAP validation using a mock community. Shown is an F1 score heatmap using eight different simulated metagnomes constructed to assess the best MASH dissimilarity cutoff across four different community structures, two different sequencing depth values, and eight different Bowtie alignment modes.

In order to evaluate the overall performance of BiG-MAP and, in particular, all the default parameters chosen as defaults, such as the Bowtie alignment mode and the MASH similarity score cutoff, we designed a mock microbial community for metagenome simulation. From the Culturable Genome Reference (CGR) genome collection (24), we randomly chose 101 CGR genomes to simulate metagenome reads from and to use as input for gutSMASH. To assess the impact of different sequencing depths (coverages of 0.5× and 0.05× to account for low- and very-low-coverage scenarios) and community structure (uniform, linear, power law, and exponential), we simulated eight different metagenomic libraries. Since the gene cluster content and their abundance levels in simulated data are known (ground truth), this allowed us to assess the recall and precision of the BiG-MAP abundance calculations using MASH dissimilarity scores ranging from 10 to 100 and the eight different alignment modes available in Bowtie across the eight different simulated data libraries. From these results we computed the F1 score or harmonic mean of precision and recall (Fig. 2), which showed that the community structure slightly affects BiG-MAP results. Moreover, since the highest F1 scores were obtained when using a MASH score cutoff (similarity) of 0.8 and using the “fast” alignment mode (end to end) of Bowtie2, we set these parameters as defaults. Still, the user is able to change them as desired by indicating this with the appropriate flag.

For orientation on the requirements and resources needed to run BiG-MAP, the time each BiG-MAP module took to run using the default settings was assessed when using as input the 3,793 gutSMASH-predicted MGCs from the 1,520 CGR collection genomes and the 78 metagenomes of the data set of Schirmer et al. (25). These analyses were run on a server with 750 GB RAM and 64 CPUs (Intel Xeon Gold 6242 CPU, 2.80GH). The details on the time and space needed to run each of the modules can be found in Table 1.

**TABLE 1:** Time and resources needed to run each BiG-MAP module on an example data set that consists of the 3,793 gutSMASH-predicted MGCs from 1,520 genomes and 78 paired-end metagenomes

BiG-MAP module	No. of CPUs	Time (approx)	Output folder size
download	1	1 day 2 h 17 min	491 GB^*a*^
family	6	4 h 2 min	4.8 GB
map	6	3 h 50 min	35 GB
analyse	1	2 min 11 s	9.3 MB

a Contains both single-end and paired-end metagenomes. Paired-end metagenomes alone occupy 249 GB.

### Analysis of the oral microbiome: revealing the presence of gene clusters associated with health and disease.

To illustrate the use of BiG-MAP in practice, we used it to analyze a data set from the human oral microbiota. The oral cavity is a natural habitat for many bacteria that reside in or on the gingival sulcus, tongue, teeth, and cheeks, among other surfaces. These bacteria take part in important processes such as initial digestion of food, but they are also associated with several oral diseases such as caries (26) and periodontitis (27). Certain oral colonists form biofilms, which can play a causal role in the development of these diseases (28). There are different functional and metabolic pathway alterations that have been associated with the onset of disease via the production of small molecules (29–32). For instance, tetramic acids produced by the caries-associated bacterium Streptococcus mutans have been linked to tooth decay (33). For this reason, in order to functionally profile these oral communities and acquire further insights into the MGCs that might be involved, we studied a data set of 47 oral microbiome samples (31) using BiG-MAP, for which paired metagenomics and metabolomics data are publicly available (see Materials and Methods sections “Assessing the *pdu* operon abundance by surveying different oral metagenomic samples” and “Evaluating the presence of the *muc* operon in caries-associated metagenomes”).

To evaluate possible molecular mechanisms underpinning caries formation, we first analyzed the available tandem mass spectrometry (MS/MS) data together with the metabolite feature abundance table using Pathway Activity Level Scoring (PALS) (34), which uses molecular families obtained using molecular networking (35) to group similar metabolites, and Pathway Level Analysis of Gene Expression (PLAGE) (36) to find differentially expressed metabolite groups between two conditions. PALS showed consistent and strong differential abundances between healthy and caries subjects in of a number of features in a metabolite group that we could annotate with polymer-like structures based on their C_3_H_4_O mass differences. With Mass Spectrometry Search Tool (MASST) searches (37) across all public data present in Global Natural Product Social Molecular Networking-Mass Spectrometry Interactive Virtual Environment (GNPS-MassIVE), we confirmed the occurrence of these differential features in various microbial, human, and environment-derived public data sets (see Materials and Methods and Text S1 for further information on the metabolomics data analysis). Based on these observations, and the size of the mass shifts observed, we concluded that these polymer-like structures could well represent molecules called polyacroleins (metabolite identification level 3—annotated compound class), which are known to spontaneously form from a component of the antimicrobial set of molecules called reuterin (38). The formation of (poly)acrolein has previously been shown to contribute strongly to the antimicrobial activity of reuterin (38). Reuterin is produced by lactobacilli from a genomic island containing a *pdu*-like operon together with a cobalamin biosynthetic gene cluster (39). Of note, acrolein is a ubiquitous compound that can be found in the human body for various other reasons as well, such as endogenous production, the ingestion of food sources, or exposure to certain environmental conditions (40). There are various known routes that can converge into the formation of acrolein, as it can be formed spontaneously from glycerol and 3-hydroxypropionaldehyde (38). Furthermore, glycerol metabolism from gut bacteria has also been found to produce this molecule (41). Typically, acrolein polymerization occurs under alkaline conditions (42); it is more likely to accumulate in saliva from healthy samples, as caries typically acidifies the oral cavity. Indeed, our results show that the possible polyacroleins are more abundant in samples of healthy volunteers. Interestingly, the presence of acrolein has been linked to inhibition of Streptococcus mutans, a well-known species of cariogenic bacteria (43, 44).

10.1128/mSystems.00937-21.1 TEXT S1 This document contains additional technical methodological details regarding the computational metabolomics analyses performed. Download Text S1, DOCX file, 0.02 MB. Copyright © 2021 Pascal Andreu et al. 2021 Pascal Andreu et al. https://creativecommons.org/licenses/by/4.0/ This content is distributed under the terms of the Creative Commons Attribution 4.0 International license.

**FIG 3:**
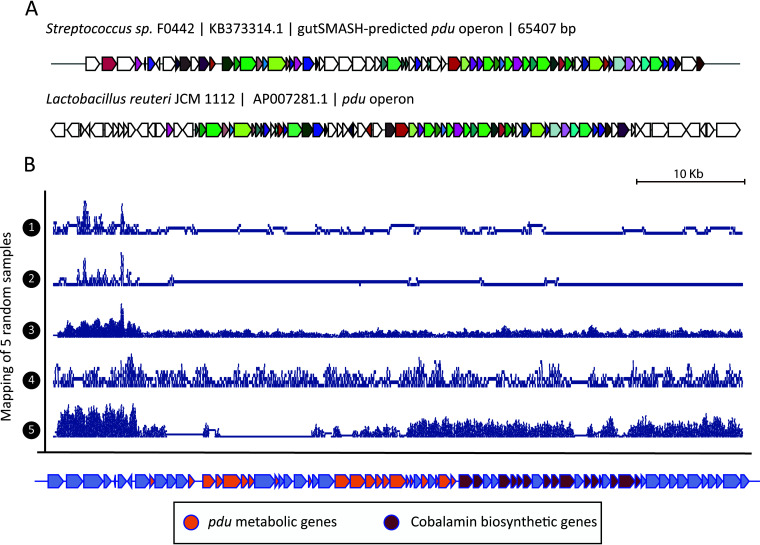
*pdu* and cobalamin operon abundance in healthy oral metagenomes. (A) MultiGeneBlast comparison between the *pdu* operon found in Streptococcus sp. F0442 by gutSMASH and the characterized one from Lactobacillus reuteri (AP007281). (B) Read coverage of five randomly chosen healthy metagenomes along the gutSMASH-predicted *pdu* gene cluster. The coverage graphs, which were plotted using the Sushi R package (version 3.5.1) (45), show that some samples (3 and 4) contain reads that cover the whole gene cluster, while in other samples, reads hardly cover the cluster (1 and 2) or cover only part of it (5).

Based on these findings, we were motivated to look for the presence of the *pdu* operon in the metagenomics samples, in order to identify candidate MGCs that might be involved in acrolein formation. To this end, we ran gutSMASH on the 1,440 genomes from the Human Microbiome Oral Database (HMOD; http://www.homd.org/) available in April 2020. Interestingly, gutSMASH identified a *pdu*-like operon in the genome of Streptococcus sp. strain F0442 that also includes a cobalamin (vitamin B_12_) biosynthetic region and is architecturally similar (cumulative BLAST bit score of 13,271) to the Lactobacillus reuteri one (Fig. 3A). Therefore, to assess the abundance of the predicted gene clusters in the oral microbiome, we used our gutSMASH run, which had predicted 3,352 gene clusters, as input for the BiG-MAP.family module, to filter out redundant MGCs. Next, the reads of 96 oral metagenomes (33 caries related, 34 healthy, 10 periodontitis related, and 19 involved in plaque development) were mapped onto the resulting 1,544 representative gene clusters using BiG-MAP.map and the counts were further normalized and parsed with BiG-MAP.analyse (see Materials and Methods section titled “Assessing the *pdu* operon abundance by surveying different oral metagenomic samples”). From this run, we found 246 gene clusters differentially abundant between groups (using a Kruskal-Wallis test) and 173 gene clusters when considering only the core region encoding the key enzymes used to detect the MGC. Among the significantly differentially abundant gene clusters observed when mapping reads to the core region, we found the *pdu* operon. While healthy samples on average have 5.62 RPKM counts/sample mapping to this gene cluster, diseased ones have 3.38 (*P* value = 0.00049 using the Kruskal-Wallis test). Of note, when using a smaller data set of only 24 healthy and 23 caries-related samples (from a single study by Aleti et al. [31]; PRJNA478018), the Streptococcus sp. F0442 *pdu* operon did not show a statistically significant differential abundance. We also evaluated the coverage of the read mapping of the core genomic region within the expanded metagenomic data sets and found that among healthy samples, not all contained this gene cluster. For instance, of 34 healthy samples in the extended data set, we found 13 that appeared not to have the Streptococcus sp. F0442 *pdu* operon (coverage below 0.5), while the rest had fairly high coverage scores, with a mean coverage value of 0.77 (selecting the samples with coverage values of at least 0.5), implying the presence of this operon or a close homologue of it (Fig. 3B). While this MGC cannot explain the difference between healthy and diseased phenotypes on its own, it might be involved in polyacrolein production and inhibition of Streptococcus mutans growth under nonacidic conditions. Since expression of the MGC would be required for metabolite production, metatranscriptomic analysis of samples where polyacrolein accumulation is observed could be an interesting follow-up analysis to test the hypothesis of the involvement of this MGC in its production. Altogether, the results of our study illustrate how BiG-MAP analysis, especially when combined with complementary omics data such as metabolomics, can generate concrete hypotheses about microbiome-associated phenotypes that can be tested in the laboratory.

**FIG 4:**
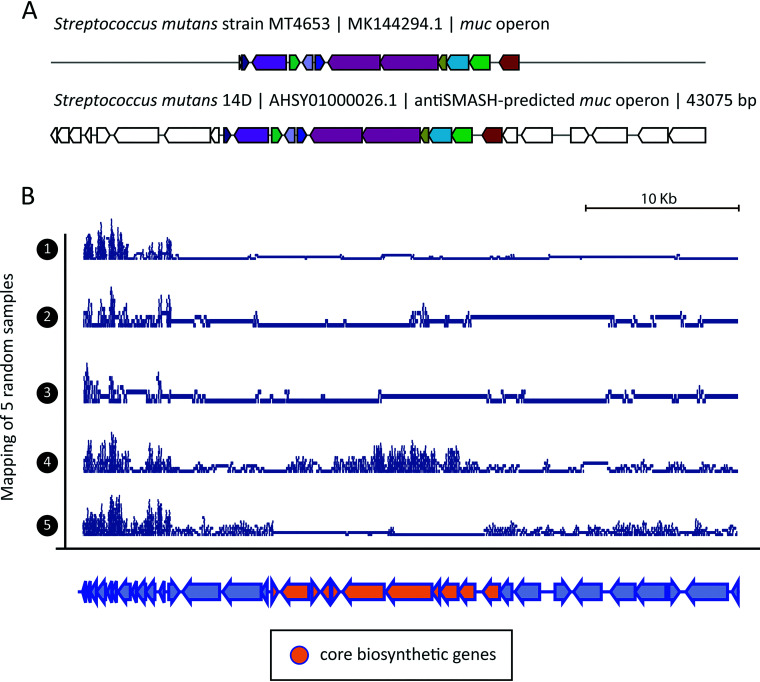
*Muc* operon overrepresentation in caries-related samples. (A) MultiGeneBlast comparison between the *muc* operon characterized from Streptococcus mutans strain MT4653.1 and the antiSMASH-predicted one from Streptococcus mutans 14D. (B) Read coverage of five randomly chosen caries-related metagenomes along the antiSMASH-predicted *muc* gene cluster. The coverage graphs, which were plotted using the Sushi R package (version 3.5.1) (45), show that despite the fact that the *muc* operon is generally not very highly covered by reads from the randomly picked samples, some seem to truly contain this operon, such as sample 4, where the core biosynthetic genes appear abundant at sufficient levels. Full data on all samples can be found in Fig. S3. It is important to note that the results found in this study come from analyzing a large collection of metagenomes that may largely differ at the community structure level. The 96 samples are not only healthy or caries-associated metagenomes but also metagenomes from patients suffering from periodontitis and samples from a study that observes how a biofilm evolves over time. Therefore, it might be that all these samples differ quite a lot in terms of MGC content but also regarding the presence of Streptococcus mutans, influencing the signal of significance reported here. All in all, our results suggest that the abundance of the *muc* operon is not very predictive for a healthy or disease state of the microbiome by itself, and other factors likely play (more) important roles.

Another example of a gene cluster that has been found relevant in the oral cavity is the *muc* operon, which has been shown to be responsible for the production of tetramic acids, which are known to inhibit the colonization of commensal bacteria in the oral cavity. This gene cluster encodes a hybrid between a polyketide synthase and nonribosomal peptide synthetase (PKS/NRPS) (33). In order to further test this association and assess whether there is a difference in abundances of the *muc* operon in the oral cavity between healthy and diseased samples, a collection of 170 Streptococcus mutans genomes collected from Tang et al. (33) and Liu et al. (46) was run through antiSMASH (10), which predicted a total of 1,849 BGCs. After obtaining 41 representative gene clusters with the BiG-MAP.family module, reads from 96 oral microbiome metagenomes (the same as for *pdu* above) were mapped onto the predicted gene clusters and further processed using BiG-MAP.map and BiG-MAP.analyse subsequently (see “Evaluating the presence of the *muc* operon in caries-associated metagenomes” in Materials and Methods). The *muc* operon from Streptococcus mutans 14D appeared differentially abundant between healthy and disease samples when using the fitZIG model (but not using the Kruskal-Wallis test), together with five other gene clusters. The *muc* operon from this strain shows high similarity to the one characterized by Tang et al. (33) (Fig. 4A). Notably, as before, when using only data from the smaller subset of samples from the study of Aleti et al. (31) (PRJNA478018), no significant difference was seen. Also, the mean read coverages of the MGC core in both groups were low, 0.274 in healthy and 0.161 in caries-associated samples, which implies a relatively low abundance of the *muc* operon in many samples and/or partial coverage of the *muc* gene cluster locus with reads mapping only to flanking regions (Fig. 4B). Nonetheless, within both groups we did see that some samples had reads mapping to the complete gene cluster, with coverage values close to 1. When filtering out samples with coverage values of <0.5, leaving only 8 samples in the healthy group and 6 in the disease group, where the MGC appears to be present, the mean coverages amount to 0.848 in healthy and 0.997 in disease. The fitZIG BiG-MAP output heatmap (Fig. S3) shows that despite the fact that the *muc* operon is significantly more abundant in diseased samples according to the test, the abundances of this gene cluster across all samples look generally quite similar. In addition, the fold change between the averages for the healthy and diseased phenotypic states is minimal, both with and without filtering. Therefore, despite finding this operon to be slightly more abundant in caries-prone samples when applying the fitZIG model, the oral microbiota from healthy donors seem to also harbor this PKS/NRPS MGC at very similar levels. Hence, the microbiota from healthy samples may have a mechanism to counteract the inhibiting effect of tetramic acids, or there might be a difference in expression of the gene cluster between healthy and diseased subjects.

10.1128/mSystems.00937-21.4 FIG S3 The *muc* operon (AHSY01000026.1.region001), predicted by antiSMASH, is significantly enriched in disease samples when applying the fitZIG model. Despite the significance, the difference in abundances between both groups is minimal, as the similar coloring depicts and the log_2_ fold change bar chart evinces. The plot shows another gene cluster, also coding for the biosynthesis of a nonribosomal peptide (NRP), found significantly enriched in disease samples. The heatmap has been produced by the BiG-MAP.analyse module using the 47 oral microbiome metagenomes. Download FIG S3, TIF file, 0.8 MB. Copyright © 2021 Pascal Andreu et al. 2021 Pascal Andreu et al. https://creativecommons.org/licenses/by/4.0/ This content is distributed under the terms of the Creative Commons Attribution 4.0 International license.

### Conclusions.

Overall, combining different omics data is a very useful approach to understand which microbes are doing what and poses a promising avenue to better understand complex biological processes. Here, we presented BiG-MAP, a command-line tool that it is able to profile the abundance and expression of a collection of gene clusters across metagenomic and metatranscriptomic data from any kind of biome, including human, plant, animal, marine, and soil microbiomes. Each of the steps in the BiG-MAP pipeline is robust, as demonstrated using a mock community. Indeed, BiG-MAP can discover interesting and relevant potential associations between genomic regions and phenotypes, which can guide experimental efforts to test MGC function. It is worth noting the usefulness of the gene cluster mapping coverage values, since they allow the user to discern between the real presence of predicted gene clusters of interest and spurious read mapping; these should always be considered together with the abundance/expression values for better interpretations of the results. Also, the associations that can be found using BiG-MAP strongly depend on the sample size and sequencing depth of the metagenome or metatranscriptome datasets. For instance, in the examples described in our study, we found that finding significant differences in the abundances of both gene clusters (the *pdu*-like operon and *muc*) depended on the size of the data set used. Both examples illustrate how users would do well to study expression or abundance values of individual samples and should not draw conclusions too quickly based on the results of a single statistical test, for which the power also depends on the number of samples available.

In addition, there are several other caveats. It is important to note that even a statistically significant difference may not be biologically important: a consistent difference with a small fold change might have limited physiological relevance. It is also important to note that differences in relative abundance may be misleading if absolute abundances of the total microbiota are different between phenotypes. Also, differences in metatranscriptomic read abundances of an MGC may be caused by differences in their (relative) metagenomic abundances (caused by differences in abundance of underlying taxa). Therefore, differences in transcript abundances may not reflect differences in gene expression levels *per se* (besides being an average of multiple taxa and cells that may act heterogeneously). If users are interested in transcriptional regulation of MGCs instead of differences in overall transcript abundance, they may therefore want to correct for this. In future versions of BiG-MAP, we hope to make it possible for users to normalize their data by absolute abundances per sample and to allow optional normalization of metatranscriptome data relative to parent taxon abundance levels inferred from metagenome data from paired samples.

Depending on the research question to be answered, the user may also consider different coverage thresholds depending on the scenario. For instance, lowering the coverage values might be needed to detect pathways from lowly abundant bacteria. Another limitation of BiG-MAP is that the family module is based on the MASH tool. In certain extreme cases, the number of predicted MGCs may exceed the maximum number of sequences that MASH (“sketch” and “dist” functions) is able to compare, leading to an error. In this scenario, the family module can be run in batches or the code can be slightly modified to manually run the MASH analysis and MASH “paste” function (more information is available in their documentation at https://mash.readthedocs.io/en/latest/) and pick up the analysis again from that step onwards.

From the BiG-MAP output folders, which include raw and processed results, it is possible to extract valuable information that helps to carefully interpret the results and to feed them into subsequent data analyses. These data include the differences within groups, coverage distribution of reads across a gene cluster, etc. In combination with detailed sample metadata, this can help provide insights into microbially derived phenotypes. Overall, we believe that BiG-MAP will help researchers solve biologically complex questions by integrative multiomics approaches, to obtain deeper insights into the relationships between microbial metabolic capacities and microbiome-associated phenotypes.

## MATERIALS AND METHODS

### Code availability.

BIG-MAP is implemented in Python 3 as a command line package. It consists of four modules: BIG-MAP.download, BIG-MAP.family, BIG-MAP.map, and BIG-MAP.analyse. The code is available at https://github.com/medema-group/BiG-MAP together with documentation on how to install BiG-MAP and its dependencies and a short tutorial on how to run it.

### BiG-MAP.download: data collection.

This module allows to retrieve sequencing data present in the SRA database using the SRA toolkit (https://github.com/ncbi/sra-tools). To initially develop, test, and validate this tool, we used an inflammatory bowel disease (IBD) cohort that contains metagenomic and metatranscriptomic data from 78 individuals, 21 suffering from ulcerative colitis (UC), 46 individuals with Crohn’s disease (CD), and 11 healthy subjects (25). These samples were retrieved using the SRA accession numbers under BioProject PRJNA389280.

### BiG-MAP.family: creating a nonredundant MGC representative collection.

The family module uses as input a directory that contains GenBank files of gene clusters identified by the antiSMASH (47) or gutSMASH (https://github.com/victoriapascal/gutsmash) algorithm. The predicted gene clusters are then subjected to a redundancy filtering step based on their mutual sequence similarity. For that, the protein sequences of the gene clusters are extracted and used as input for MASH (20), which creates sketches from the raw sequences. The sketches are then used to calculate the distances between sequences using MASH dist. The resulting tab-delimited file with the pairwise distance comparisons is used to group gene clusters with above a 0.8 default similarity cutoff (Fig. 2). Next, to pick the best representative of each group, medoids are computed (see formula below). For this, a distance matrix is created comparing all distances between pairs of gene clusters; the one with minimal cumulative distance value is picked as representative of that group. Additionally, the selected gene clusters are subjected to another round of clustering using BiG-SCAPE (19), to cluster gene clusters into GCFs at a 0.3 distance cutoff (default value), from which a random representative is picked.

$$x_{\text{medoid}}={\text{argmin}}_{y\in \{x_{1}\;,\;x_{2},\ldots \;x_{n}\}}\overset{n}{\underset{i=1}{\sum }}d(y,\;x_{i})$$

If metatranscriptomes are used in the BiG-MAP.map module, an additional step is performed to set an expression baseline. For this, the protein sequences of the genomes whose gene clusters form the nonredundant representative gene cluster collection are scanned using hmmsearch (hmmsearch version 3.1b2) for five housekeeping-coding proteins: DNA gyrase A (PF00521), DNA gyrase B (PF00204), recombinase A (PF00154), DNA-directed RNA polymerase A (PF01000), and DNA-directed RNA polymerase B (PF00562). The selection of these Pfam domains was based on the findings by Rocha et al. (48) that the genes for these housekeeping proteins show highly stable expression across samples. Next, the gathered protein sequences are also used as queries in the mapping module to align metatranscriptomic reads to gene clusters.

### BiG-MAP.map: mapping reads to a nonredundant gene cluster collection.

This module relies on Bowtie2 (21) (version 2.3.4.3) to align reads to a given sequence. From the reference gene cluster sequences selected by the medoid calculation, Bowtie index files are created. Next, Bowtie2 aligns reads to these index files, using the fast alignment mode by default. The resulting alignment is stored in SAM format and converted to BAM format to later be parsed by SAMtools (49) (version 1.9). The alignments are then sorted by their leftmost coordinates; the aligned reads are counted and either summed by GCF or averaged over the GCF size. Later, the corrected raw counts are converted to TPM (transcripts per kilobase million) counts and consecutively to RPKM (reads per kilobase million) counts to account for sequencing depth.

Another functionality that was added in this module was to compute the read coverage of each gene cluster using the coordinates in the sorted BAM files. To do so, the sorted alignment files are converted to bedgraphs using BEDtools (50) (v2.28.0), which allow estimating the number of covered bases for each cluster (coverage) by subtracting the number of noncovered bases (*ncb*) to the length of each cluster (*cl*) as indicated in the formula below.

$$\text{coverage}=\frac{\mathrm{cl}\;-\;\mathrm{ncb}}{\mathrm{cl}}$$

The same procedure is followed to compute the RPKM counts and the coverage of the core genes within a gene cluster, which strictly considers the core metabolic genes within each gene cluster. This information is taken from the antiSMASH/gutSMASH (or any other SMASH-related algorithm) GenBank output files that flag the key coding genes that are needed for the synthesis of a given molecule. Once the core genes are identified, their alignment information is retrieved using SAMtools. Next, in the same manner as RPKM values are computed for the whole gene clusters, reads aligned to the core region are extracted, counted, and corrected to finally get the RPKM counts. To perform the coverage calculation, the locations of the core genes are extracted from the bedgraph to evaluate the coverage.

### BiG-MAP.analyse: normalization of RPKM counts and finding differentially expressed/abundant MGCs.

In order to account for sparse high-throughput sequencing, RPKM values are normalized using cumulative sum scaling (CSS) from the R Bioconductor package MetagenomeSeq (22). BiG-MAP offers two different statistics to account for differentially abundant/expressed gene clusters: the parametric zero-inflated Gaussian distribution mixture model (ZIG model) and the nonparametric Kruskal-Wallis test. ZIG model values are adjusted with log_2_ fold change that ultimately helps fitting the model to a log-normal distribution; thus, when the abundance/expression values are expected to follow normal distribution, the ZIG model is more appropriate to use. Alternatively, the Kruskal-Wallis test can be run on the normalized RPKM counts, which allows assessing whether the distribution of ranks for one group significantly differs from the distribution of ranks for the other group. Additionally, false-discovery rate (FDR) correction is applied to correct for multiple hypothesis testing. Finally, heatmaps are produced to visualize the results using the Seaborn Python package (https://github.com/mwaskom/seaborn).

### Testing BiG-MAP performance using a mock community.

To test BiG-MAP performance, 101 bacterial genomes were randomly chosen from the CGR collection (24) (BioProject number PRJNA482748). Thus, the gutSMASH-predicted MGCs from each genome were used as ground truth (https://github.com/victoriapascal/gutsmash, version 0.8; github commit stamp 569e860). Next, paired-end reads were generated with a mean read length of 100 bp from the 101 CGR bacterial genomes using Grinder v0.5.3 (51). Two different read coverage thresholds were used (0.5× and 0.05×) in combination with four different community structures: uniform, linear, power law, and exponential. Both the MGCs and the simulated reads were used as input for BiG-MAP, which was run ranging the MASH similarity thresholds between 10 and 100% in intervals of 10% along the eight different Bowtie2 alignment modes. From each individual run, true-positive, false-positive, and false-negatives rates were calculated to evaluate the precision and recall, which was ultimately used to compute the harmonic mean of precision and recall, also known as the F1 score. The results were plotted in a heatmap using the ComplexHeatmap package in R (52).

### Assessing the *pdu* operon abundance by surveying different oral metagenomic samples.

To find possible leads on metabolic perturbances between healthy and caries-related samples, the processed mass spectra (MGF format) and metabolomics feature tables from Aleti et al. (31) were downloaded from GNPS-MassIVE (35) accession number MSV000081832 to perform reanalysis. Feature-based Molecular Networks (53) were run using GNPS release version 21 (https://gnps.ucsd.edu/ProteoSAFe/status.jsp?task=ef4f64542ab24a7fb0802ceacbcfa071, https://gnps.ucsd.edu/ProteoSAFe/status.jsp?task=9c95754d1fdc42b4a43b16919c398ecd). The resulting molecular family information together with the metabolite feature tables and sample information (metadata) were loaded into PALS (https://pals.glasgowcompbio.org/app/) (34) to identify metabolite families differing in activity between 25 healthy and 24 caries-related samples. From the results, three out of seven candidate metabolites in one differentially expressed molecular family showing clear different abundance patterns between healthy and caries samples were further examined using GNPS MASST (https://masst.ucsd.edu) (37), the ChemCalc MF finder (54), and PubChem (55), leading to the putative annotation of polyacrolein-related metabolites in healthy samples, which may be produced from a *pdu*-like operon that requires the presence of the cobalamin biosynthetic genes (see supplemental material for further information).

For the analysis of the *pdu* operon and its presence in the oral microbiome, 1,440 oral bacteria genomes were downloaded from the HOMD collection (http://www.homd.org/?name=GenomeList&link=GenomeList&type=all_oral). Next, these genomes were used as input for gutSMASH (version 0.8). The comparison between the two *pdu* operons from Lactobacillus reuteri (AP007281) and Streptococcus sp. F0442 (GCA_000314795.2) was done using MultiGeneBlast (56). Next, all predicted clusters were used as input for the BiG-MAP family module. At the same time, the oral metagenomics data sets were downloaded using the BiG-MAP.download module by providing the SRA accession numbers associated with BioProject numbers PRJNA478018, PRJNA396840, and PRJNA398963. Once the metagenomes were downloaded, BiG-MAP.map was run using the output of the family module and the metagenomic reads in FASTQ format. Finally, the RPKM counts were normalized, processed, and visualized using BiG-MAP.analyse.

### Evaluating the presence of the *muc* operon in caries-associated metagenomes.

AntiSMASH was used to predict BGCs from a total of 170 Streptococcus mutans genomes reported by Tang et al. (33) and Liu et al. (46). Within the predicted BGCs, the *muc* operon was found and compared to the *muc* operon characterized by Hao et al. (57) using MultiGeneBlast (56). The predicted BGCs were then used as input for the BiG-MAP.family module. Both the representative BGCs and metagenomic reads were then used as input in the subsequent BiG-MAP.map mapping module using the metagenomes from the following three BioProjects: PRJNA478018, PRJNA396840, and PRJNA398963. Finally, the raw mapping counts were normalized and further processed and visualized using BiG-MAP.analyse.

### Data availability.

The supporting information for this article can be found in the supplemental material of this article and in the Zenodo repository (https://zenodo.org/record/5054950) with the following doi: 10.5281/zenodo.5054950. The metabolomics data used for reanalysis are available from GNPS-MassIVE accession number MSV000081832.
