# Supplementary figures and images for: BiG-MAP: an Automated Pipeline To Profile Metabolic Gene Cluster Abundance and Expression in Microbiomes
Source: mSystems. 2021 Sep 28;6(5):e00937-21. doi: 10.1128/mSystems.00937-21 (PMC8547482; doi:10.1128/mSystems.00937-21)

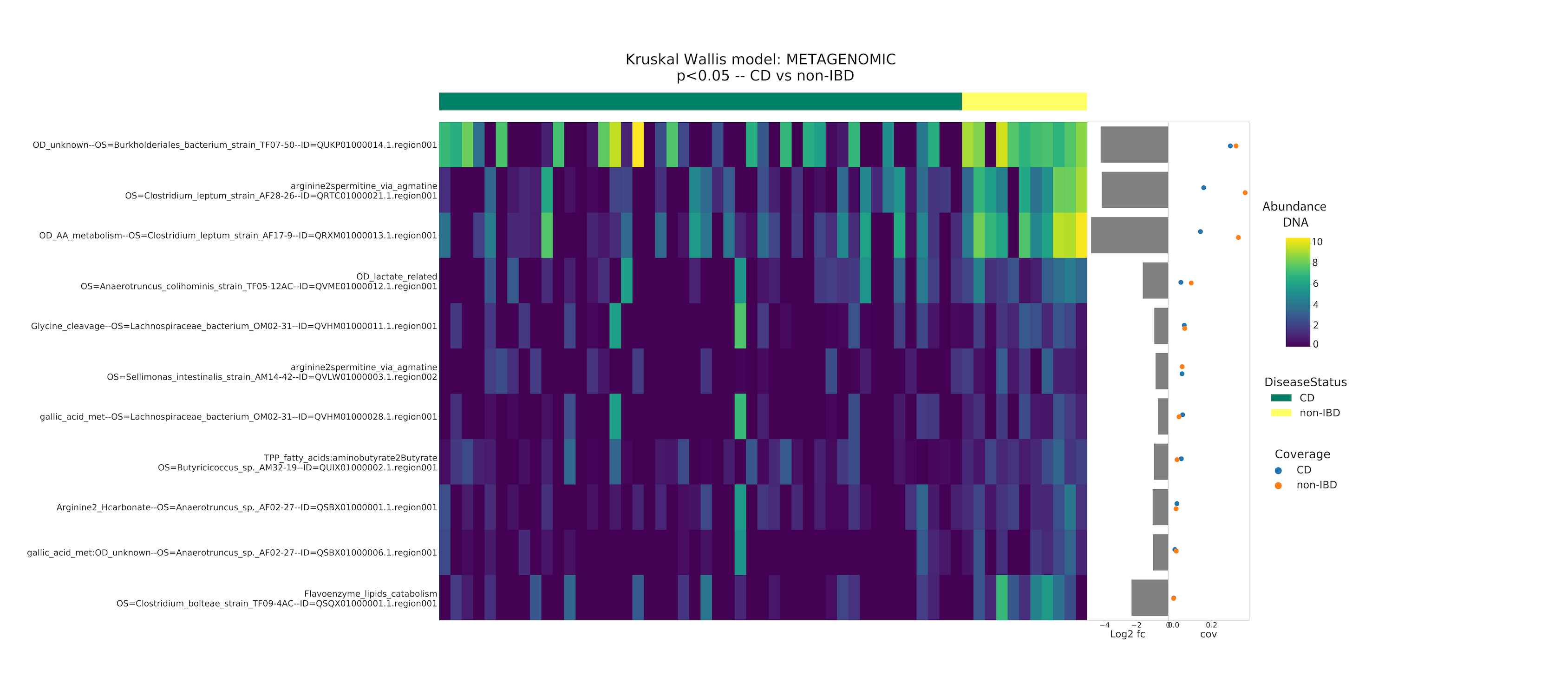

Supplement: FIG S1 [file msystems.00937-21-sf001.tif]

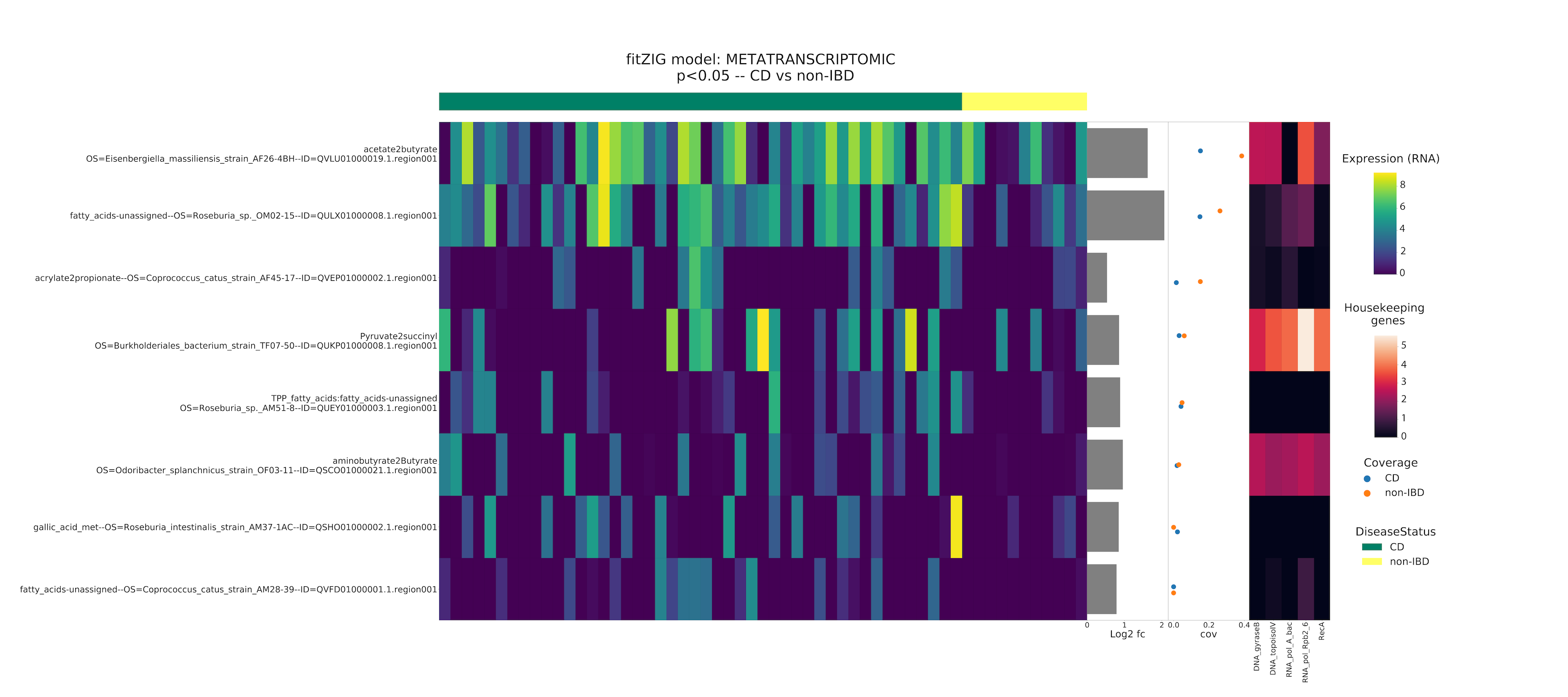

Supplement: FIG S2 [file msystems.00937-21-sf002.tif]

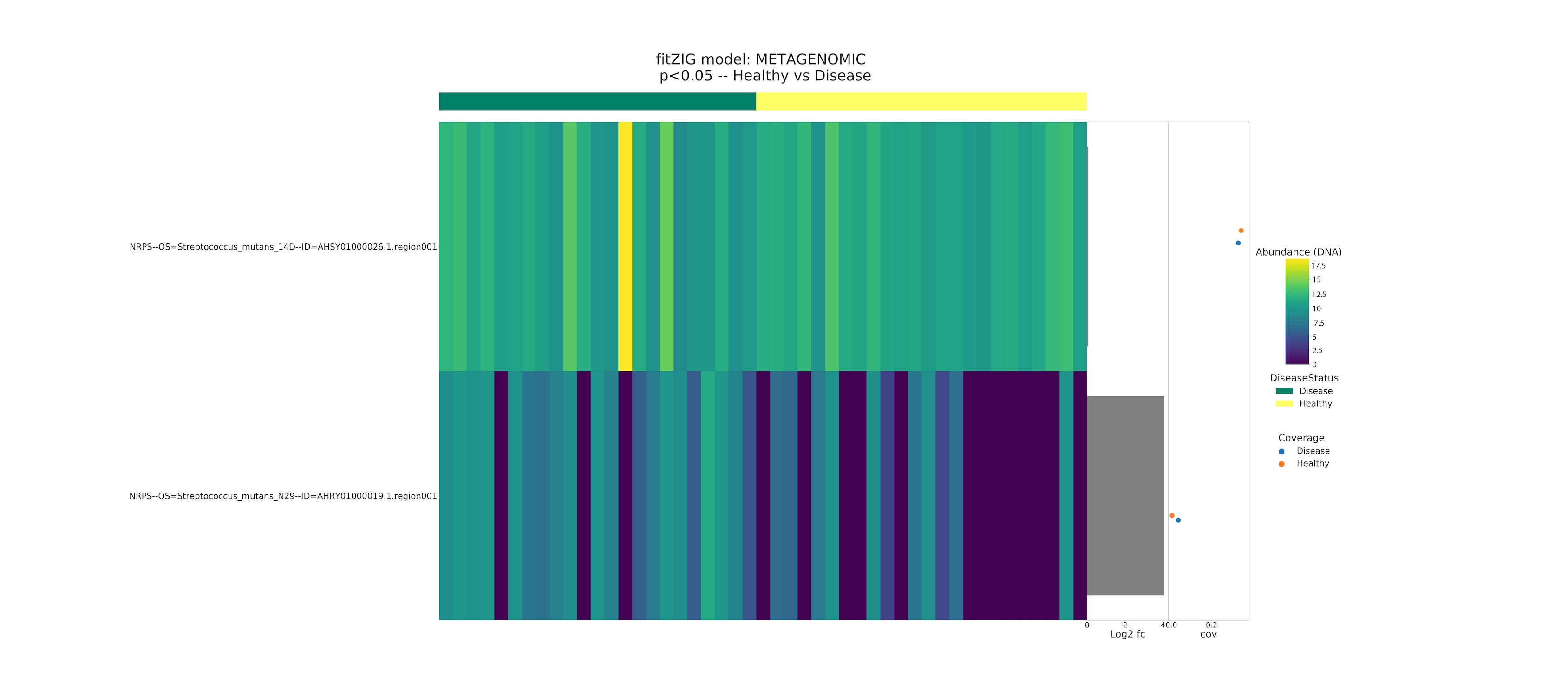

Supplement: FIG S3 [file msystems.00937-21-sf003.tif]
