# Supplementary material for: BiG-MAP: an Automated Pipeline To Profile Metabolic Gene Cluster Abundance and Expression in Microbiomes
Source: mSystems. 2021 Sep 28;6(5):e00937-21. doi: 10.1128/mSystems.00937-21 (PMC8547482; doi:10.1128/mSystems.00937-21)
Supplement: TEXT S1 [file msystems.00937-21-t0001.docx]

**Supplementary Text 1 for: BiG-MAP: an automated pipeline to profile metabolic gene cluster abundance and expression in microbiomes**

Victoria Pascal Andreu*^1^, Hannah E. Augustijn*^1^, Koen van den Berg^1^, Justin J. J. van der Hooft^1^, Michael A. Fischbach^2^, Marnix H. Medema^#1^

1. Bioinformatics Group, Wageningen University, Wageningen, The Netherlands
2. Department of Bioengineering and ChEM-H, Stanford University, Stanford, USA

**Metabolomics analyses**

GNPS Molecular Networking was done as follows:

A molecular network was created with the Feature-Based Molecular Networking (FBMN) workflow on GNPS (<https://gnps.ucsd.edu>). The data was filtered by removing all MS/MS fragment ions within +/- 17 Da of the precursor m/z. MS/MS spectra were window filtered by choosing only the top 6 fragment ions in the +/- 50 Da window throughout the spectrum. The precursor ion mass tolerance was set to 0.02 Da and the MS/MS fragment ion tolerance to 0.02 Da. A molecular network was then created where edges were filtered to have a cosine score above 0.65 and more than 5 matched peaks. Further, edges between two nodes were kept in the network if and only if each of the nodes appeared in each others respective top 10 most similar nodes. Finally, the maximum size of a molecular family was set to 100, and the lowest scoring edges were removed from molecular families until the molecular family size was below this threshold. The analogue search mode was used by searching against MS/MS spectra with a maximum difference of 200.0 in the precursor ion value. The library spectra were filtered in the same manner as the input data. All matches kept between network spectra and library spectra were required to have a score above 0.7 and at least 6 matched peaks.

GNPS Molecular Networking job of mass spectrometry data: <https://gnps.ucsd.edu/ProteoSAFe/status.jsp?task=9c95754d1fdc42b4a43b16919c398ecd>

MASST searches were performed for a number of features that showed typical C3H4O mass differences using the default settings. A single spectrum search was completed using the online workflow (<https://ccms-ucsd.github.io/GNPSDocumentation/>) on the GNPS website (<http://gnps.ucsd.edu>). The data was filtered by removing all MS/MS fragment ions within +/- 17 Da of the precursor m/z. MS/MS spectra were window filtered by choosing only the top 6 fragment ions in the +/- 50 Da window throughout the spectrum. The precursor ion mass tolerance was set to 2.0 Da and a MS/MS fragment ion tolerance of 0.5 Da. The library spectra were filtered in the same manner as the input data. All matches kept between input spectra and library spectra were required to have a score above 0.7 and at least 6 matched peaks.

Feature ID 3779, precursor m/z 680.4799:

<https://gnps.ucsd.edu/ProteoSAFe/result.jsp?task=71831f592f27496faf16c62bc16b1b69&view=view_all_datasets_matched>

Feature ID 153, precursor m/z 722.4900:

<https://gnps.ucsd.edu/ProteoSAFe/result.jsp?task=79bc074e6e274f05ac4d3a273c50681a&view=view_all_datasets_matched>

Feature ID 126, precursor m/z 663.4528 :

<https://gnps.ucsd.edu/ProteoSAFe/result.jsp?task=8742b10dda2a4e29bc405fdc34aa1aa6&view=view_all_datasets_matched>
